# Significance of platinum distribution to predict platinum resistance in ovarian cancer after platinum treatment in neoadjuvant chemotherapy

**DOI:** 10.1038/s41598-022-08503-7

**Published:** 2022-03-16

**Authors:** Kaname Uno, Nobuhisa Yoshikawa, Akira Tazaki, Shoko Ohnuma, Kazuhisa Kitami, Shohei Iyoshi, Kazumasa Mogi, Masato Yoshihara, Yoshihiro Koya, Mai Sugiyama, Satoshi Tamauchi, Yoshiki Ikeda, Akira Yokoi, Fumitaka Kikkawa, Masashi Kato, Hiroaki Kajiyama

**Affiliations:** 1 Department of Obstetrics and Gynecology, Nagoya University Graduate School of Medicine, 65, Tsurumai-cho, Showa-ku, Nagoya, Aichi 466-8550 Japan; 2 Division of Clinical Genetics, Department of Laboratory Medicine, Graduate School of Medicine, Lund University, Lund, Sweden; 3 Department of Occupational and Environmental Health, Nagoya University Graduate School of Medicine, Nagoya, Japan; 4 Spemann Graduate School of Biology and Medicine, University of Freiburg, Freiburg, Germany; 5 Department of Obstetrics and Gynecology Collaborative Research, Graduate School of Medicine, Bell Research Center, Nagoya University, Nagoya, Japan

**Keywords:** Cancer, Medical research, Oncology

## Abstract

Most patients with ovarian cancer experience recurrence and develop resistance to platinum-based agents. The diagnosis of platinum resistance based on the platinum-free interval is not always accurate and timely in clinical settings. Herein, we used laser ablation inductively coupled plasma mass spectrometry to visualize the platinum distribution in the ovarian cancer tissues at the time of interval debulking surgery after neoadjuvant chemotherapy in 27patients with advanced high-grade serous ovarian cancer. Two distinct patterns of platinum distribution were observed. Type A (n = 16): platinum accumulation at the adjacent stroma but little in the tumor; type B (n = 11): even distribution of platinum throughout the tumor and adjacent stroma. The type A patients treated post-surgery with platinum-based adjuvant chemotherapy showed significantly shorter periods of recurrence after the last platinum-based chemotherapy session (p = 0.020) and were diagnosed with “platinum-resistant recurrence”. Moreover, type A was significantly correlated with worse prognosis (p = 0.031). Post-surgery treatment with non-platinum-based chemotherapy could be effective for the patients classified as type A. Our findings indicate that the platinum resistance can be predicted prior to recurrence, based on the platinum distribution; this could contribute to the selection of more appropriate adjuvant chemotherapy, which may lead to improves prognoses.

## Introduction

Approximately 70% of ovarian cancer cases are diagnosed at an advanced stage with peritoneal dissemination^1^. High-grade serous ovarian carcinoma (HGSOC) is the most common subtype of ovarian cancer. Primary debulking surgery followed by adjuvant chemotherapy is the standard treatment for ovarian cancer, and neoadjuvant chemotherapy followed by interval debulking surgery (NAC-IDS) is another alternative^2^. Most cases of HGSOC are sensitive to initial platinum-based agents, and with such treatment > 70% of HGSOC patients can achieve complete remission^2,3^. However, nearly 80% of the patients experience recurrence and become resistant to platinum-based agents, which is related to poor long-term survival^3,4^.

In clinical settings, recurrence within 6 months after the last dose of platinum-based chemotherapy is referred to “platinum-resistant recurrence.” The interval between the date of the last platinum use and the date of relapse detection is called the platinum-free interval (PFI)^1,4^. Whether or not to use platinum-based agents at the time point of a patient’s recurrence depends on the patient's PFI. The problem with this strategy is that it allows for the diagnosis of platinum resistance only at the time of tumor growth^1,4,5^. Moreover, the PFI is often inconsistent with true platinum resistance, because the PFI does not always reflect molecular mechanisms^5,6^. Novel biomarkers of platinum resistance are needed so that more appropriate drugs can be used for HGSOC earlier in adjuvant treatment, which could prolong the patients’ survival and minimize adverse effects caused by platinum-based agents^6^.

**Figure 1:**
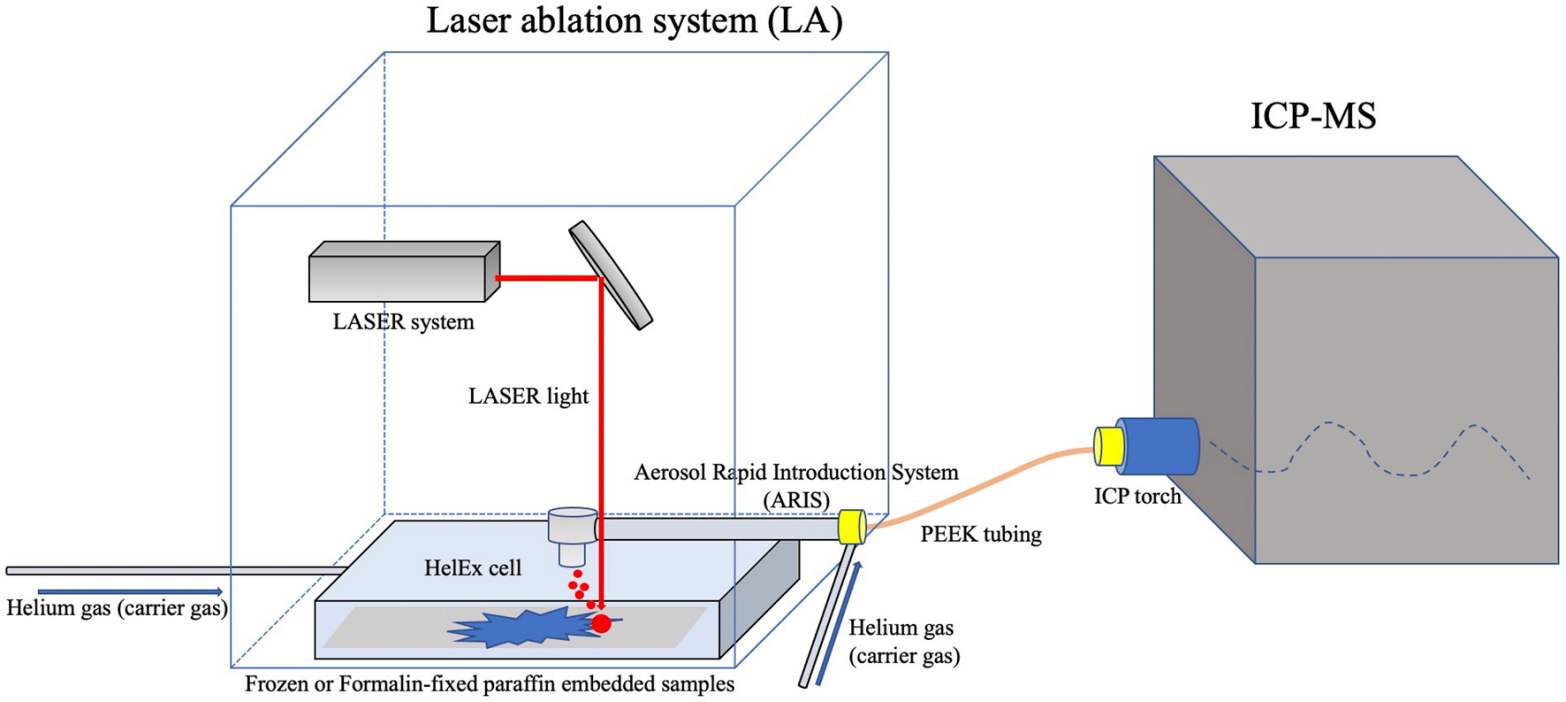
Representative LA-ICP-MS image. FFPE samples inserted into a HelEx cell box are ablated by the focused laser. The ablated samples are carried through the aerosol rapid introduction system (ARIS) to the ICP-MS.

The intracellular platinum concentration has been reported to be correlated with the platinum sensitivity of lung, bladder, and ovarian cancer cell lines^7–10^. Inductively coupled plasma mass spectrometry (ICP-MS) can detect the platinum concentration in dissociated cells with the use of nitric acid. It has not been possible to distinguish between the platinum distributions in a tumor and the platinum distribution in adjacent stroma, but we speculated that the recently introduced method of laser ablation ICP-MS (LA-ICP-MS), which uses a focused laser ablation system combined with ICP-MS^11^ (Fig. 1), may be useful for distinguishing these platinum distributions. LA-ICP-MS is used to identify the distribution of trace elements in tissue, and a few investigations using LA-ICP-MS have identified the platinum in cancer patients in whom platinum-based agents had been used^12–16^. However, to our knowledge, no reports on the LA-ICP-MS analysis of platinum distributions to clarify drug resistance and prognosis have been published. We conducted the present study to investigate the usefulness of performing LA-ICP-MS to identify platinum distribution in order to predict of platinum resistance and cancer prognosis at the time of NAC-IDS.

## Results

### Classification of NAC-IDS samples using LA-ICP-MS

**Figure 2:**
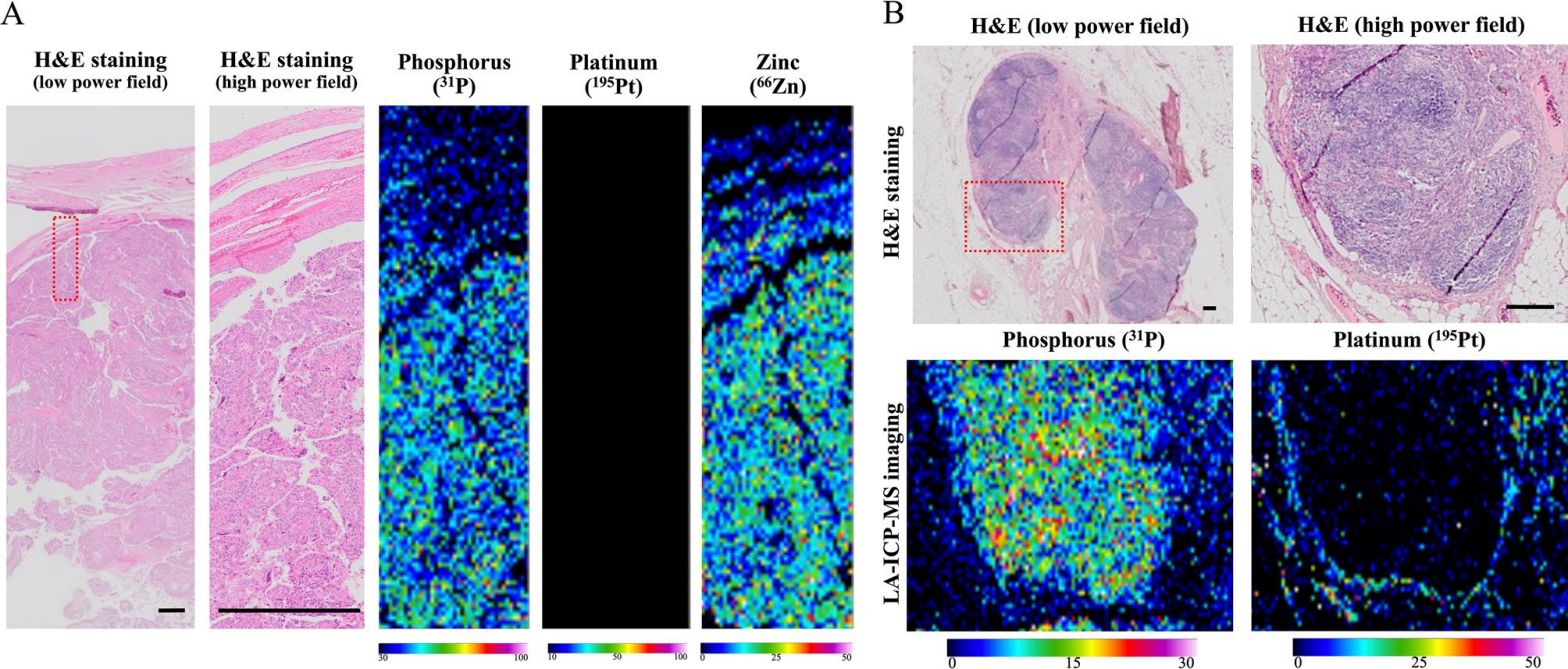
Phosphorus and platinum distributions in clinical samples using laser ablation ICP-MS (LA-ICP-MS). (**A**) Representative LA-ICP-MS images of high-grade serous ovarian cancer in primary debulking surgery as a negative control of the platinum and phosphorus and zinc distribution in tumor. Scale bar: 200 μm. (**B**) Images of phosphorus and platinum distributions in a clinically platinum-resistant recurrent ovarian tumor. Platinum was not present in the tumor but had accumulated in the borderline area of the tumor and stroma. Scale bar: 200 μm.

We analyzed primary debulking surgery specimens from patients who had not undergone treatment with platinum-based agents as a negative control using LA-ICP-MS (n = 5). The operational condition is summarized in Table 1. iQuant 2+ software is used to create images of trace elements^17^. The images created with the iQuant 2+ software could be compared with the hematoxylin and eosin (H&E)-stained samples (Fig. 2A). The LA-ICP-MS revealed a high levels of phosphorus and zinc in tumor areas in the negative controls, but platinum was not present in either the tumor or the stroma (Fig. 2A).

**Table 1:** LA-ICP-MS operational conditions.

**Laser ablation system**	
Type	CETAC LSX-213 G2 +
Wavelength	213 nm
Pulse duration	< 5 ns
Laser repetition rate	20 Hz
Laser spot size	20 μm
Laser energy	5.1 J/cm^2^ (25%)
Laser scan speed	20 μm/s
Laser beam geometry	circular
Helix He gas flow	0.225 flow rate
Innercup He gas flow	0.20 flow rate
**Inductively coupled plasma mass spectrometry**	
Type	Agilent 7700X
Plasma power	1550 W
Carrier Ar gas glow	1.07 flow rate
Collision cell gas	He
Collision cell gas flow	5.0 flow rate
Cone	Ni
Acquisition element	^13^C, ^31^P, ^195^Pt, ^66^Zn

*C* carbon, *He* Helium, *LA-ICP-MS* laser ablation inductively coupled plasma mass spectrometry, *Ni* nickel, *P* phosphorus, *Pt* platinum, *Zn* zinc.

As positive controls, we next evaluated clinically platinum-resistant recurrent ovarian cancer tissues. This patient was treated platinum-based agents after the recurrence because PFI was > 6 moths at the time of recurrence. However, her recurrent tumor had shown no response to platinum-based treatment, therefore we resected the solitary tumor in abdomen. After the operation, her tumor was considered to be clinically platinum resistant, and regimen was changed to non-platinum-based agents. To our surprise, in this case, platinum was not detected in the tumor area but had accumulated in the adjacent stroma (Fig. 2B). Furthermore, multiple sections of tumor tissues were analyzed by LA-ICP-MS. There was little platinum accumulation inside tumor. And platinum was accumulated in the stromal areas (Suppl. Fig. S1). To be effective for tumor suppression, platinum should enter tumor cells and bind tumor’s DNA. Therefore, we hypothesized that platinum is not able to exist in the tumors that do not respond to platinum agents. And we also hypothesized that true platinum sensitivity can be diagnosed by platinum distribution at the tumor margins before recurrence.

**Figure 3:**
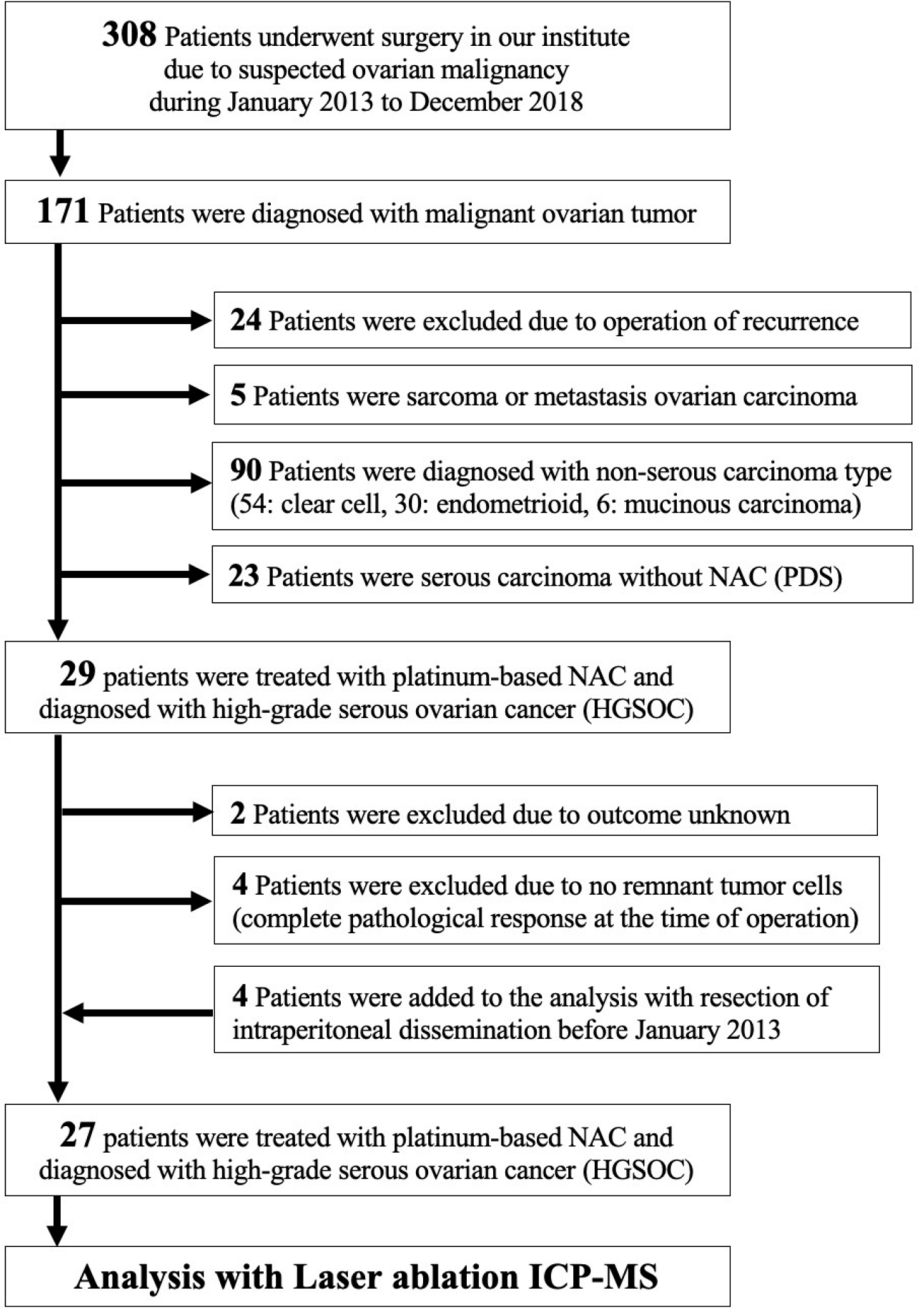
Flowchart of patient inclusion. We enrolled the total of 308 patients who were suspected of having ovarian malignancy and underwent surgery at our institution during the period from January 2013 to December 2018. Among of them, 171 patients were diagnosed with malignant ovarian cancer. The 27 patients with HGSOC at stage III or IV according to the revised 2014 FIGO staging system who were treated with NAC-IDS including platinum-based agents were analyzed in this study.

**Figure 4:**
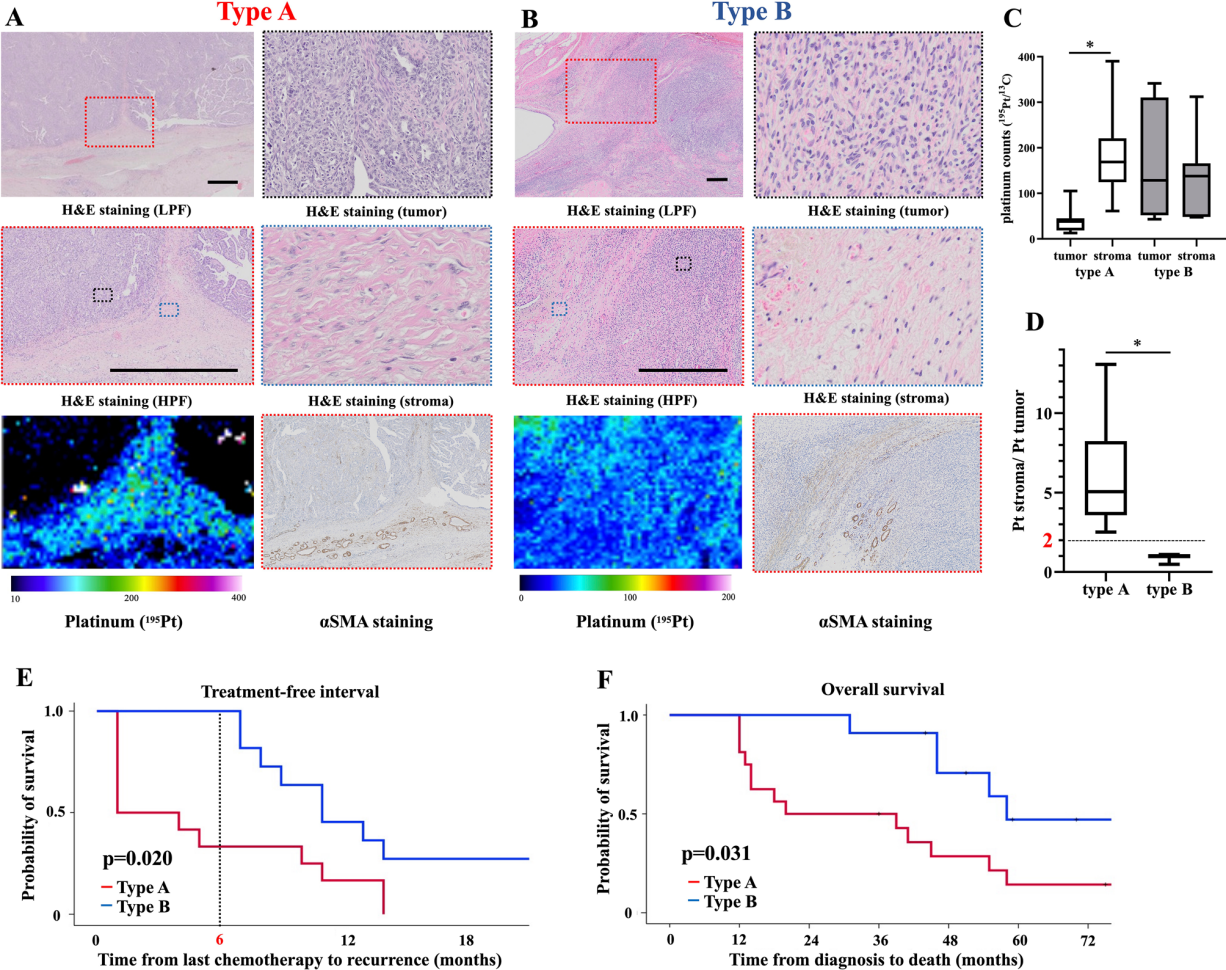
Platinum distribution and survival in the patients with HGSOC. (**A**) Representative images of types A. In type A, there is little platinum accumulation in the tumor but accumulation in stroma. Scale bar: 200 μm. (**B**) Representative images of type B. In type B, platinum was also present in the tumor. Scale bar: 200 μm. (**C**) ^195^Pt counts in tumor and stroma in types A and B. The ^195^Pt counts in the type A tumors were significantly lower than those in the stroma. (**D**) The platinum counts in the stroma in the type A cases were over twofold those of the the tumors. (**E**) Kaplan–Meier analysis of the treatment-free interval (TFI) in both groups in of patients treated with platinum-based agents in adjuvant chemotherapy. The type A patients developed significantly earlier recurrence, and most of them were diagnosed with platinum-resistant recurrence (p = 0.020). (**F**) Kaplan–Meier analysis of the two groups’ overall survival (OS). The type A patients had significantly shorter survival compared to the type B patients (*p* = 0.031).

We then analyzed the HGSOC samples which were treated with platinum regimens before operation. A total of 27 NAC-IDS patients were enrolled (Fig. 3). We have classified these patients into two groups according to the distribution of platinum by LA-ICP-MS. Type A refers to specimens with low platinum counts in the tumor but with the high platinum counts in the adjacent stroma, whereas type B refers to specimens with the comparable platinum counts both in the tumor and adjacent stroma. Representative images of each type are shown in Fig. 4A,B. We could divide stroma from tumor in a sample with high magnified H&E staining as shown in Fig. 4A,B. And to detect stroma area, immunohistochemistry of αSMA was also useful in some cases (Fig. 4A,B, Suppl. Fig. S2). A few cases with difficulty to distinguish the tumor with the stroma were analyzed using p53 or Ki-67 staining to detect tumor area correctly. Using IgG conjugated with colloidal gold (Au) as second antibody, LA-ICP-MS could detect the positive p53 or Ki-67 staining area, which meant tumor area. In the type A patients, platinum did not exist in the area of Au (Suppl. Fig. S3).

The platinum counts in type A specimens were significantly higher in the adjacent stroma compared to the tumor (Fig. 4C,D). The type A pattern of platinum distribution was relatively the same as that of the clinically platinum-resistant tumor as a positive control shown in Fig. 2B. By contrast, in the type B, no apparent difference of platinum counts was observed between the tumor and the adjacent stroma (Fig. 4C,D). The difference of platinum accumulation in the border of the tumor and adjacent stroma has much information of platinum distribution. Therefore, we selected for LA-ICP-MS analyses only the border of the tumour tissues because that area can be suitable area to analyze by LA-ICP-MS and to know the platinum resistance.

### The type of platinum accumulation at the adjacent stroma was related to platinum-resistant recurrence and poor prognosis

Table 2 summarizes the background of the 27 patients with HGSOC. The NAC cycles and duration from the last NAC cycle to IDS were 3–10 cycles and 23–53 days, respectively. In most cases, the CA125 level was decreased from diagnosis to surgery. The observational periods were 37–103 months.

**Table 2:** Patients’ background.

Characteristics	Number or median	Percentage or range
Total patients	27	
Age at diagnosis, years (median)	61	41–75
**Clinical stage (number)**		
IIIC	18	66.7%
IVA	1	3.7%
IVB	8	29.6%
NAC cycle, times (median)	6	3–10
Duration from last NAC to IDS, days (median)	35	23–53
Complete surgery (number)	18	66.7%
**Adjuvant chemotherapy (number)**		
Platinum-based	21	77.8%
Non-platinum based	4	14.8%
No adjuvant therapy	2	7.4%
CA125 level, U/mL (median)	1881	142–17,434
**At diagnosis**		
Presurgery	26	8.2–587
Recurrence (number)	24	88.9%
Death (number)	19	70.3%

*NAC* neo-adjuvant chemotherapy, *IDS* interval-debulking surgery.

Of the 27 cases, 16 (59.2%) were classified into type A and the other 11 (40.7%) were type B. The patient’s characteristics by group are presented in Table 3. No significant differences between the two types were observed in age, NAC courses, duration from NAC to IDS, CA125 level at diagnosis, or the rate of complete surgery. Tumor regression after chemotherapy was significantly higher in the patients with type B (type A: 50% vs type B: 70%, p = 0.029). In terms of recurrence, none of the type B patients in type B experienced recurrence within 6 months from the last platinum-based drug. Conversely, nine of the 16 (56.3%) type A patients experienced recurrence within 6 months (Treatment-Free Interval [TFI] < 6 months). TFI was defined that interval from last chemotherapy to recurrence or death. Among the 16 type A patients, though 11 were treated with platinum-based agents in adjuvant chemotherapy, 4 were treated with non-platinum-based chemotherapy based on a clinical diagnosis of platinum resistance from pathological findings. The other one rejected additional adjuvant chemotherapy. Eight of the nine (88.9%) type A patients treated with the same platinum-based chemotherapy after IDS relapsed within 6 months. Three of the four type A patients treated with non-platinum-based agents after IDS had a TFI > 6 months. Although the Kaplan–Meier analysis did not reveal a significant difference in TFI between the type A and B patients (Suppl. Fig. S4A, p = 0.160), it identified a significantly difference between the type A and B in only patients who were treated post-surgery with platinum-based agents (Fig. 4E, p = 0.020).

**Table 3:** Patients characteristics in both groups.

	Type A (n = 16)	Type B (n = 11)	p-value
Age at diagnosis, years (median, range)	61.5 (41–75)	60.0 (41–71)	0.394
NAC course, times (median, range)	5.5 (3–9)	6.0 (3–10)	0.121
Duration from NAC to IDS, days (median, range)	35.0 (23–50)	36.0 (24–53)	0.645
CA125 at diagnosis, U/mL (median, range)	3208 (161–17,434)	1640 (142–6220)	0.368
CA125 presurgery, U/mL (median, range)	58.6 (8.2–587)	15.0 (11–34)	0.023
Complete surgery (number, %)	10/16 (62.5%)	8/11 (72.7%)	0.692
Tumor regression rate, % (median, range)	50 (10–90)	70 (30–95)	0.029

*NAC* neo-adjuvant chemotherapy, *IDS* interval-debulking surgery.

With respect to OS, all but one of the 11 type B patients lived for > 3 years after diagnosis, whereas eight of the 16 type A patients died within 3 years after diagnosis, demonstrating that the type A patients had a significantly worse prognoses (Fig. 4F, p = 0.031). Among the group of type A patients, those treated with non-platinum-based agents in adjuvant chemotherapy tended to have longer TFIs (Suppl. Fig. S4B, p = 0.071).

**Figure 5:**
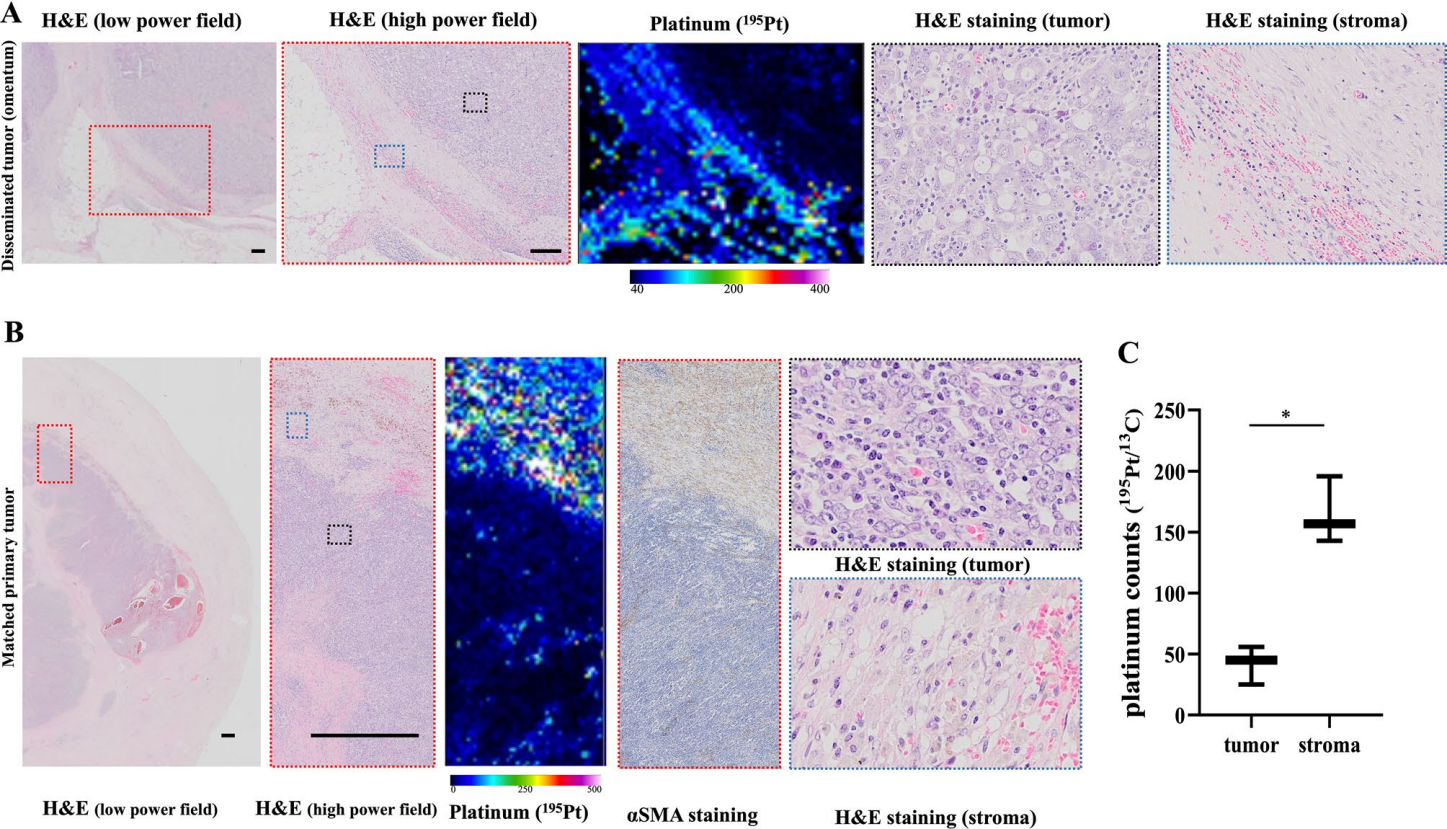
Platinum distribution of the primary tumors and disseminated tumors. (**A**) Representative images of an abdominal dissemination in a type A case. Platinum was accumulated in a marginal area in tumor and stroma and little platinum was present in the tumor. Scale bar: 200 μm. (**B**) The platinum distribution of a matched primary tumor (ovary) of a type A patient. Little platinum existed in the tumor compared to the stroma. Scale bar: 200 μm. (**C**) The platinum counts in the tumor and stroma in disseminated tumors (n = 4).

Lastly, we have analyzed the distribution of platinum in the peritoneal metastasis lesions. Only omentum metastasis of type A patients were included. These patient’s clinical information is summarized in Supplementary Table S1. The accumulation of platinum in the adjacent stroma and little platinum in the tumor were also recognized in the matched disseminated tumors, which is the same distribution as that of the primary tumors (Fig. 5A–C, n = 4).

## Discussion

We obtained the novel findings that (1) LA-ICP-MS clearly revealed the platinum distribution in NAC-IDS samples from patients with ovarian cancer, and (2) there are two characteristic patterns of platinum distribution that indicate platinum resistance and prognosis prior to recurrence. The platinum distribution identified by LA-ICP-MS can be a reasonable biomarker of true platinum resistance at the time of NAC-IDS in patients with advanced ovarian cancer and can provide useful information for the selection of the appropriate chemotherapy after operation.

Platinum resistance, which is currently defined based on the PFI at the time of recurrence^1,5,18^, is a major problem in ovarian cancer treatment^1,3,5^. The definition using the time of recurrence means that platinum resistance cannot be determined until recurrence is observed, and this is why platinum-based drugs are often used until disease progression (Suppl. Fig. S5). In this study, we analyzed NAC-IDS specimens using LA-ICP-MS and observed that platinum could be detected in the cancer tissues and that the distribution of platinum could be quite different between the tumor and the adjacent stroma. The results of our analyses demonstrated that the pattern of platinum distribution was significantly associated with platinum resistance and long-term survival. Based on these results, we speculate that it may be possible to predict the future acquisition of platinum-resistant recurrence at the time of IDS and to identify patients with a poor prognosis due to acquired platinum resistance.

Our LA-ICP-MS analysis revealed that the patients with the type A platinum pattern showed a distinctive distribution of platinum, with an accumulation in the adjacent stroma of the cancer tissues (Fig. 4A). Various studies of platinum resistance have demonstrated that one of the most reliable mechanisms is a reduction in platinum uptake or an enhanced extracellular efflux of platinum through transporters^1,5,9,19,20^. However, the earlier studies were not able to directly confirm the distribution of platinum in cancer tissues. The inability of platinum to enter to a tumor or efflux from a tumor leads to its accumulation at the tumor margins, and the platinum distribution in the type A pattern with accumulation at the tumor margin is consistent with these mechanisms. To the best of our knowledge, this is the first study to identify the characteristic patterns of platinum distribution in cancer tissues. The platinum distribution revealed by LA-ICP-MS could be a promising biomarker of platinum resistance in the treatment of ovarian cancer.

In this study, we could detect platinum in all the samples, even 23 to 53 days after administration of platinum-based agents (Fig. 4A,B). According to the studies of pharmacokinetics of platinum-based agents, platinum concentrations in plasma decrease rapidly because of its lipophilicity^21,22^. The mean half-life of free platinum in plasma ranged from 6 to 25 min^21,23,24^. On the other hand, platinum of various tissues were detected at the same concentration for 10 days after cisplatin injection^22,25^. In erythrocytes, a platinum concentration peak occurring within the 3 h following the end of infusion. Then, concentration of platinum decline slowly, with a mean half-life ranging from 29 to 50 days^21^. The mean platinum concentration of plasma or cells are varied between studies because of sampling frequency, study duration, kinds of platinum-based agents, and assay methods. There is no study addressing the half-time of platinum in cancer tissue using LA-ICP-MS. Our findings demonstrated that platinum still existed even 50 days after platinum-based agents.

Our analyses also showed that the TFI was significantly shorter in the type A patients compared to the type B patients, and we suspect that this significant difference is indicative of acquired platinum resistance in the patients treated with platinum-based agents in adjuvant chemotherapy (Fig. 4E, p = 0.020). In our type A patients, the rate of recurrence at < 6 months, which is the current clinical definition of platinum-resistant recurrence, was 56.3% (9 of 16 patients), and eight of those nine patients (88.9%) were treated with the platinum-based agents even in adjuvant chemotherapy. This result may indicate both anegative effect of using the same platinum-based agents and the potential efficacies of non-platinum-based agents on the prolongation of the TFI in type A patients, who have potentially acquired platinum resistance.

The OS was also significantly worse in the type A group compared to the type B group (Fig. 4F, p = 0.031). This may be because platinum-based drugs are often used for type A patients in the current clinical settings since no tool exists to determine platinum resistance at the time of IDS. Actually, six of the eight (75.0%) type A patients treated with the same preoperative platinum-based agents in postoperative treatment died within 2 years. The method of visualizing platinum distribution by LA-ICP-MS may serve as a better biomarker of platinum resistance and contribute to a better treatment strategy for patients with acquired platinum resistance.

We observed that the platinum distribution in the peritoneal dissemination had the same pattern as that in the matched primary tumors (Fig. 5A,B). The primary site (ovaries) is removed at the time of IDS, but the disseminated tumor sometimes remains after operation. Over 70% of patients with advanced ovarian cancer experience recurrence in the abdomen, which is the most common cause of death among ovarian cancer patients: therefore, even if the tumor is thought to be completely removed, most patients are likely to be treated with additional anticancer drugs after surgery^1,5,19^. As noted above, the LA-ICP-MS results indicated that the pattern of platinum distribution in the disseminated lesions was the same as that of the primary tumors.

**Figure 6:**
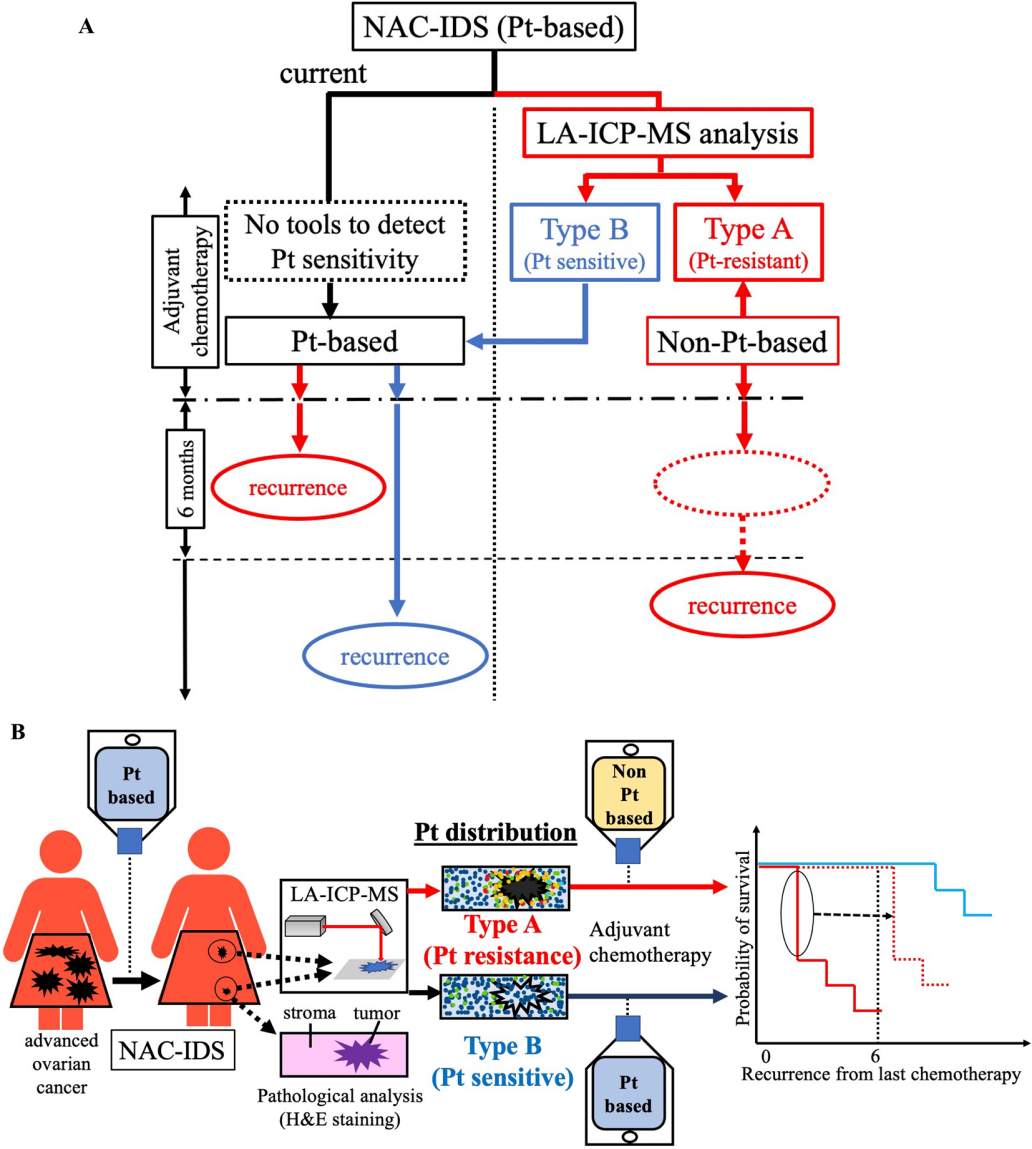
New ovarian cancer treatment strategy using LA-ICP-MS. (**A**) LA-ICP-MS revealed platinum resistance in NAC-IDS samples before recurrence. Since type A patients have a significant worse prognosis when the same platinum-based agents are used in adjuvant chemotherapy, we suggest a change to non-platinum-based adjuvant chemotherapy for patients classified as type A in order to improve their disease progression and prognosis and to decrease the side effects of inefficient platinum-based agents. (**B**) Graphic abstract in this study. In samples from patients with high-grade serous ovarian cancer who have undergone neoadjuvant chemotherapy followed by interval debulking surgery, laser ablation–inductively coupled plasma mass spectrometry (LA-ICP-MS) reveals the two distinct patterns of platinum distribution of the tumor and the adjacent stroma: decreased platinum in the tumor and accumulation in the adjacent stroma (named ‘type A’ herein), which is related to platinum resistance with short latency to recurrence and poor prognosis compared to the type B in which the platinum distribution is similar in the tumor and stromaBy using this technique at the time of surgery, we can select appropriate chemotherapy for each patient, which can improve their prognoses.

Analyses of NAC-IDS specimens using LA-ICP-MS can provide us with reliable information that could be used to determine postoperative ovarian cancer treatment. We suggest a new ovarian cancer treatment strategy using LA-ICP-MS (Fig. 6A). By using LA-ICP-MS analysis after platinum-based agents, we can identify patients who are likely to have a poor prognosis in current clinical settings and then choose the appropriate anti-cancer agents. This strategy can improve patients’ prognoses. Figure 6B shows the graphic abstract in this study.

There are some limitations of this study. Only 27 patients from a single institution were included, and underlying biases in our analyses could not be controlled due to the retrospective study design. Prospective larger-scale studies are needed to verify whether treatment strategies that incorporate LA-ICP-MS prolong the prognosis. In this study, we have analyzed only the tissues of ovarian cancer, although platinum-based agents are widely used in various types of tumors. In addition, the areas that can be analyzed by LA-ICP-MS are limited because it takes to analyze by LA-ICP-MS. The present analysis was performed using tissues merely from the border of the tumor and stroma.

In conclusion, the distribution of platinum was clearly visualized by LA-ICP-MS. The duration to subsequent recurrence was significantly shorter in the type A group, who showed a lower concentration of platinum in the tumor than in the adjacent stroma. LA-ICP-MS is a powerful tool to predict platinum resistance and long-term survival. In the future, the analysis of NAC-IDS specimens using LA-ICP-MS can offer guidance toward more appropriate adjuvant anticancer treatment and can change the treatment strategy for patients with advanced ovarian cancer.

## Patients and methods

### Ovarian cancer samples for LA-ICP-MS analysis

Our study was approved by the Ethics Committee at Nagoya University (No. 2017-0497). We enrolled the total of 308 patients who were suspected of having ovarian malignancy and underwent surgery at our institution during the period from January 2013 to December 2018. Among of them, 171 patients were diagnosed with malignant ovarian cancer. Ninety patients were excluded due to other histological type of malignant ovarian cancer. The 27 patients with HGSOC at stage III or IV according to the revised 2014 FIGO (International Federation of Gynecology and Obstetrics) staging system who were treated with NAC-IDS including platinum-based agents were analyzed in this study. At least two pathologists agreed to diagnose in our hospital, and the authors also have ability to diagnose of ovarian cancer and detect the tumor area. Two patients were excluded due to outcome unknown, and four patients were excluded because of a lack of remnant tumor cells. For the analysis of both primary and disseminated lesions, four patients whose tumor existed both within an ovary and omentum metastasis were included (Fig. 3). We collected and retrospectively analyzed the patients’ clinical information including age, NAC cycles, duration from last NAC to IDS, the values of cancer antigen (CA), 125 at diagnosis and before IDS, tumor regression after chemotherapy, the interval from last chemotherapy to recurrence or death (defined as the treatment-free interval; TFI), and overall survival (OS), defined as the time from diagnosis to death, was retrospectively collected. Tumor regression rate were estimated by the area of viable tumor and the rate of necrosis area. These areas were detected by H&E staining. The intraperitoneal disseminated lesions in IDS in four cases were included in the analysis. Tissue samples removed by debulking surgery without the use of platinum-based agents were used as a negative controls. For positive controls, we used clinically platinum-resistant recurrent samples that were resistant to platinum-based agents after recurrence, then resected by surgery.

### LA-ICP-MS

An CETAC LSX-213 G2+ laser ablation system (Teledyne Cetac, Omaha, NE, USA) was used; it is equipped with a frequency quintupled 213-nm Quantel Ultra Compact Q-Switched Nd: YAG MIL-SPEC Laser and coupled to a7700X ICP-MS instrument (Agilent Technologies, Santa Clara, CA) (Fig. 1). The ovarian cancer tissue sections were ablated by a focused laser beam with energy of 5.1 J/cm^2^ and scan speed was 20 µm/s in our LA-ICP-MS scanning system reported before^11^. The laser ablated the samples at a 20-µm-dia. spot on the surface. The detailed instrumental parameters are summarized in Table 1.

### Sample preparation and classification of LA-ICP-MS images

Consecutive sections, 4-to 6-µm thickness, from formalin-fixed paraffin-embedded (FFPE) cancer tissue were analyzed. H&E staining was performed to identify the marginal area of the cancer tissue where the tumor and the adjacent stroma meet. The adjacent section was used for LA-ICP-MS analysis. The counts of ^13^C, ^31^P, ^195^Pt and ^66^Zn were analyzed. ^31^P, ^195^Pt and ^66^Zn were adjusted ^13^C counts to make a per-specimen correction for the amount of laser-evaporated tissue. Localized images of these trace elements were generated by the iQuant 2+ software program^17^. We identified the stroma or tumor area by comparing images of trace element and consecutive H&E staining. H&E slides were scanned using a virtual slide microscope VS120-S5 (Olympus, Tokyo, Japan). Immunostaining of Ki-67 and p53 was performed using anti-Ki-67 rabbit monoclonal antibody (CST Ki67 #9027) and anti-p53 rabbit monoclonal IgG (CST, p53, #2527S) to detect tumor area in some cases. These staining were performed according to manufacturer’s manuals. Anti-rabbit IgG conjugated with 12 nm colloidal gold (Jackson Lab., #111-205-144) was used as second antibody. The count of ^197^Au was analyzed by LA-ICP-MS followed by iQuant 2+ for localized images of Ki-67 or p53. To detect the stroma area, immunohistochemistry of αSMA (Abcam. αSMA, ab124964) was performed in several samples. The counts of ^195^Pt and ^31^P were performed as an average of 20 randomly selected spots form the tumor and the stromal sites.

Classification of the specimens, type A and type B, was made by the following criteria based on the platinum distribution. Type A refers to specimens with low platinum counts in the tumor but with the high platinum counts in the adjacent stroma. Type B refers to specimens with the comparable platinum counts both in the tumor and adjacent stroma. We defined type A as the pattern in which the platinum-counts ratio of stroma to tumor is > 2.0, and type B was the pattern in which the corresponding ratio is ≤ 2.0. Metastasis of omentum were analyzed as the same way of primary tumor. Each platinum distribution was also divided into type A or B according to the same definition described above. We also compared platinum distribution pattern between the primary tumors and matched disseminated tumors in the type A patients.

### Statistical analyses

Non-normally distributed data are reported as the medians and minimum and maximal ranges. Categorical variables are presented numerically and as percentages. Non-normally distributed data were analyzed with non-parametric tests. A Kaplan–Meier survival analysis was used for the analyzes of the TFI and OS. The probability of significance was set at *p* < 0.05. All statistical analyzes were performed using the IBM SPSS software package (ver.27.0, IBM Corp., Armonk, NY, USA).

### Ethics approval and consent to participate

Our study was approved by the Ethics Committee at Nagoya University (No. 2017-0497). The IRB issued a waiver for written ‘informed’ consent because of no additional sample collection. The study was performed in accordance with the Declaration of Helsinki.

## Supplementary Information

Supplementary Table S1.

Supplementary Figures.

## Abbreviations

HGSOC: High-grade serous ovarian carcinoma
IDS: Interval debulking surgery
LA-ICP-MS: Laser ablation inductively coupled plasma mass spectrometry
NAC: Neoadjuvant chemotherapy
OS: Overall survival
PFI: Platinum-free interval
Pt: Platinum
TFI: Treatment-free interval
