# Supplementary material for: Significance of platinum distribution to predict platinum resistance in ovarian cancer after platinum treatment in neoadjuvant chemotherapy
Source: Sci Rep. 2022 Mar 16;12:4513. doi: 10.1038/s41598-022-08503-7 (PMC8927415; doi:10.1038/s41598-022-08503-7)
Supplement: Supplementary file 1 — Supplementary Table S1. [file 41598_2022_8503_MOESM1_ESM.docx]

**Supplementary Table S1.** Clinical information in patients with metastasis

| **Case** | | 1 | 2 | 3 | 4 |  |  |  |
| --- | --- | --- | --- | --- | --- | --- | --- | --- |
| **Age at diagnosis, yrs** | | 62 | 48 | 53 | 57 |  |  |  |
| **NAC course, times** | | 6 | 4 | 5 | 5 |  |  |  |
| **Duration from NAC to IDS, days** | | 35 | 23 | 30 | 33 |  |  |  |
| **CA125 level, U/mL** | |  |  |  |  |  |  |  |
| At diagnosis | | 8,605 | 161 | 4,124 | 1,676 |  |  |  |
| Presurgery | | 237 | 8.2 | 505 | 102 |  |  |  |
| **Completion of IDS** | | Complete | Complete | Complete | Suboptimal |  |  |  |
| **Type of platinum distribution** | |  |  |  |  |  |  |  |
| Primary tumor (ovary) | | A | A | A | A |  |  |  |
| Intraperitoneal dissemination (omentum) | | A | A | A | A |  |  |  |
|  | NAC: neo-adjuvant chemotherapy, IDS: interval-debulking surgery. | | | | | |  |  |
